# A conceptual framework of supply chain resilience towards sustainability through a service-dominant logic perspective

**DOI:** 10.1016/j.heliyon.2023.e13901

**Published:** 2023-02-20

**Authors:** Hakim Manurung, Gatot Yudoko, Liane Okdinawati

**Affiliations:** School of Business and Management, Institut Teknologi Bandung (ITB), Bandung, Indonesia

**Keywords:** Service-dominant logic, SDL, Supply chain, Supply chain resilience, SCR, Sustainability

## Abstract

The supply chain of every company is essential to its overall operation. Global disruption has an unanticipated and immediate impact on supply chains across all industrial sectors. The supply chain must, therefore, demonstrate resilience in order to resist such upheaval because it is dynamic and extremely vulnerable to global uncertainties. Moreover, it must not only return to its initial form but also prove able to achieve steady, sustainable performance. To do so, supply chain activities necessitate value co-creation between stakeholders. One means of achieving this objective is through the application of service-dominant logic (SDL) whose eleven core premises define the behaviour, governance, and consequences of service-based exchange. The novelty of the research reported here lies in the mapping and review frameworks of current supply chain resilience towards sustainability (SCRTS) from an SDL perspective. This study represents the first to combine an SDL perspective and SCRTS since no formula has yet been devised to address this combination of factors. This study proposes the application of a conceptual framework of SCRTS from an SDL perspective by involving selected premises, resource integration, institutional structures, and collaboration.

**Type of Paper:**

Conceptual Framework.

## 1 Introduction

The supply chain of each company is essential to its overall operation and should, therefore, have a competitive advantage. In an era of disruption, a company's ability to compete depends on the competitiveness of its supply chain [1]. Unfortunately, being a component of the main chain, the supply chain is extremely vulnerable to global disruption [2] which affects those of any industry unexpectedly and with unforeseen notice. Hence, the supply chain requires continual resilience and sustainable performance in order to respond to such disruption [3]. characterize supply chain resilience (SCR) as a process of continually adapting, evolving, and transforming in response to the dynamic multi-scale feedback between the multitude of interconnected organizations, institutions, and social and ecological systems constituting supply chain elements. The phrase SCR has been commonly employed since approximately 2004 and scholars agree that, in essence, it is concerned with the ability of a company to return to its original condition [4]. However, since the supply chain is dynamic and highly sensitive to global disruptions, SCR requires not only a return to its initial form but also the capacity to demonstrate steady, sustainable performance [5]. To achieve sustainable performance, supply chain activities necessitate value co-creation between stakeholders formed on the basic premise of service dominant logic (SDL), specifically resource integration, institutional arrangements, and stakeholder collaboration. Since being introduced by Ref. [6], SDL has been adopted by many disciplines, including supply chain theory, and has evolved as a theoretical framework from a linear exchange of tangible resources and embedded value to a more systemic interchange of intangible resources and value co-creation [7].

SDL comprises eleven core premises that define the behaviour, governance, and consequences of service-based exchange. The current SDL framework demonstrates that a dynamic, multi-dimensional system perspective more aptly reflects the structural aspects of service exchange. Creating supply chain resilience towards sustainability (SCRTS) through SDL constitutes a new movement within supply chain studies, a fact indicative of SCR being viewed as a service supply chain. However, because of the operant and operand resource exchange, the only premises adopted in this study are those of resource integration, institutional arrangement, and collaboration.

The main purpose of this study is to establish how SDL perspectives influence SCM strategy through past studies by proposing a conceptual framework. From an SDL perspective, the supply chain must consider more than just arrangements relating to the supply of goods. It must also consider all activities viewed as service exchange. The research literature has particularly contributed to the body of knowledge regarding supply chain management when the SDL concept is applied to activities. As this study presents a conceptual framework, it is intended to serve as a guide for future research involving analysis of real-life data relating to a specific industry. However, the framework can be adjusted based on the actual phenomenon.

Supply chain research has produced numerous concepts that can be applied within any industrial sector, although it tends to be focused more on practical strategies incorporating traditional concepts such as goods dominant logic [8]. Several studies have discussed the broader implementation of SDL within supply chains to achieve resilience and sustainability. The value co-creation framework proposed by Ref. [9] is applied in their study to assess the body of previous research. These authors highlight the need to address study gaps in the field and explore how the paradigm shift introduced by SDL in relation to market exchanges affects the traditional supply chain view. A content analysis of the supply chain services listed in the sources consulted is also included in the systematic literature review undertaken by Ref. [10]. They attempt to analyze these services from the various perspectives of SDL to further the existing theory. Other reviews integrating an SDL interface with a supply chain cover various salient topics of interest, including innovation [11], general SCM by identifying SDL as one of the key trends [12], business models [13], and servitization [14]. Despite the expanding body of research, no systematic literature reviews currently exist that compile, summarize, and describe the research being undertaken in these two fields, particularly that with a business marketing and supply chain focus which highlights resilience leading to sustainability.

By performing a thorough evaluation of the literature on this topic, this study seeks to bridge this gap and advance the fields of both SCM and SDL by addressing the following research questions. Firstly, what insights developed by the SDL discipline can be implemented within the supply chain body of knowledge? Secondly, which antecedents of SDL and SCRTS can be constructed within a conceptual framework? Thirdly, based on the literature review, what represents an appropriate framework for SDL impact on SCRTS that may be applied to any industry?

The structure of this study consists of the following sections: a literature review; an analysis of the theory connecting SDL and SCRTS which identifies the antecedents and combines the foundation premises (FPs) of SDL with the supply chain. The description of the methodological approach adopted is followed by an outline of the study's conceptual framework.

## 2 Literature review

Service science, such as SDL, is a new concept involved in supply chain resilience research [15]. enrich the supply chain concept by incorporating an SDL perspective into the supply chain which, in turn, shifts the paradigm from good dominant logic to service dominant. The SDL perspective focused on how inter-company collaboration may lead to the creation of superior value propositions through mutual learning, thereby enhancing their overall knowledge, skills, and abilities ([16,6]). According to Ref. [17], supply chain management entails not only the delivery of goods but also an exchange of services. Knowledge, integration, customer engagement, connections, and innovation are all aspects of exchanges between supply chain stakeholders which are promoted by SDL ([18,19,20]). There are eleven SDL foundational premises (FPs) and five axioms [21] that can be summarized in the following tenets of service exchanges:

• Every economy constitutes a service economy. With greater specialization and outsourcing, the nature and role of service (singular) are only now becoming more evident.
• Resource integrators include all economic and social actors. Value is produced by a group, and for this to occur, inputs (resources) must be combined.
• Actor-generated institutions and institutional structures coordinate value co-creation.

### 2.1 Bridging the concepts of SDL and SCRTS

The main idea behind adopting SDL within SCRTS is that the former employs a process perspective in which various actors collaborate to co-create value, particularly during the utilization process, by integrating resources and exchanging underlying services [15] Scholars have identified SDL premises possessing relationships between the variables or enablers of SCRTS. An examination of the literature highlights nine essential ideas: value co-creation, operand and operant resources, collaboration, networks and ecosystems, resource integration, partnerships, strategic advantages, learning, and communication ([22,20]). The enabler of SCRTS found that the SCR dimension is separated into three categories: proactive signifying pre-disruption or preparedness; reactive featuring during-disruption or adaptability; and recovery indicating post-disruption or adjustment ([5,23]). Proactive features comprise redundancy, robustness, attentiveness, awareness integration, and visibility, while collaboration, information exchange, agility, adaptability, and speed serve as reactive enablers. Leadership, financial stability, re-configuration, re-design, and adaptability constitute the enablers of recovery. However, this study will only review the pertinent factors that support SDL and SCRTS.

A supply chain is more than just a means of moving items, irrespective of whether or not this fact is recognized. Eleven FPs intended to establish a framework for a service-centered mindset encapsulate the logic of service dominance [20]. have recognized since the initial introduction of the foundational premises that a number of the original FPs could be derived from others. As a result, they identified five of the eleven expanded FPs as particularly foundational, essentially the axioms of SDL, as described in Table 1.

**Table 1:** SDL's axioms and foundational premises [20].

AXIOM	Code	Premises
Axiom 1	FP1	Service is the fundamental basis of exchange.
	FP2	Indirect exchange masks the fundamental basis of exchange.
	FP3	Goods are distribution mechanisms for service provision.
	FP4	Operant resources are the fundamental source of competitive advantage.
	FP5	All economies are service economies. Service (singular) is only now becoming more apparent with increased specialization and outsourcing.
Axiom 2	FP6	The customer is always a co-creator of value.
	FP7	The enterprise cannot deliver value, but only offer value propositions.
	FP8	A service-centered view is inherently customer-oriented and relational.
Axiom 3	FP9	All economic and social actors are resource integrators.
Axiom 4	FP10	Value is always uniquely and phenomenologically determined by the beneficiary.
Axiom 5	FP11	Value co-creation is coordinated through actor-generated institutions and institutional arrangements.

In keeping with the tenets of SDL-FP3 which assert that goods are distribution mechanisms for service provision, they are also a means of service delivery. Numerous researchers have viewed the supply chain through the service eco-system paradigm since [17] first introduced the concept of SDL to scholars. Within this service exchange, value is a constellation of resources integrated by all actors involved in providing the service, with co-created value for the customer always being uniquely defined by the end-user [17]. Resources can be either operand or operant, with competitive advantage and differentiation potential stemming from the latter which can be defined as the knowledge, skills, and capabilities of the actors involved in service delivery.

[10] explain the concepts of service and services, respectively, as follows: “An act or performance offered by one party to another. Although the process may be tied to a physical product, the performance is essentially intangible and does not normally result in ownership of any of the factors of production and services are economic activities that create value and provide benefits for customers at specific times and places, as a result of bringing about a desired change in or on behalf of the recipient of the service.” These definitions of service and services supplement SDL's axioms. According to Ref. [24], service is the application of resources (primarily knowledge and skills) for the benefit of another person or organization (the beneficiary). All businesses constitute service businesses because SDL requires interaction between the service provider and the recipient [24]. Moreover, it is imperative that employees, managers, suppliers, customers, and other stakeholders collaborate to integrate resources (combining or aligning assets) for mutual value creation (producing benefit for all participants). In addition to general conceptualization efforts, special consideration is afforded service supply chains from a service-dominant perspective ([25,10,26])and a performance-based logistics perspective ([25,10,27,28]).

Several studies of the interface between SDL and the supply chain adopt opposing positions [29]. conducted a literature review using the framework for value co-creation developed in the course of their study. They intended to investigate how the paradigm shift that SDL introduces to market exchanges affects the traditional supply chain perspective while, in addition, identifying research gaps in the field that subsequent researchers have found fruitful. For the purposes of theory development [10], included a content analysis of the supply chain services mentioned in the selected literature and attempted to analyze them from the various dimensions of SDL. Other reviews, including a supply chain and an SDL interface focused on different main topics of interest such as innovation [11], supply chain recognition of SDL as one of the influential trends [12], and servitization [30].

According to Ref. [31], SDL links supply chains to partnerships, value networks, service providers, and value generators. If all supply chain layers such as the producer-customer layer, supplier-producer layer, and supplier's supplier-supplier layer adopt the service eco-system scenario, the entire supply chain forms a complex value network that deals with partnership, service provision, and value creation. In other words, the SDL-based supply chain may maximize each supply chain member's economic, social, and environmental performance [32]. Even though an ever-increasing body of literature has been produced on this topic, no systematic reviews exist that collect, summarize, and describe ongoing research in these two areas, with a specific focus on SDL and SCRTS.

### 2.2 Resource integration

“Cooperation” and “collaboration” are words occasionally employed interchangeably with the term “integration” to refer to either the internal or external variety. Utilization and integration of value network operant resources enhance the ability of the whole chain to achieve competitive advantage. Internal integration refers to that occurring between departments within an organization ([33,34,35]) while its external counterpart involves integration with partners outside the company. External integration manifests itself, for example, in joint planning or information sharing to achieve the shared objectives of supply chain partners ([34,36,35]).

The degree to which an organization works strategically with its supply chain partners and cooperatively controls intra- and inter-organizational operations is referred to as supply chain integration. Its goal is to establish efficient and effective flows of products and services, information, money, and options at a low cost and rapid pace that maximize value to customers [37]. [32] identified internal integration as exerting the greatest influence on supply chain resilience, exceeding that of customer and partner integration. Customer integration was found to have three completely mediating impacts on the links between internal integration and service performance; internal integration and service quality; and logistics collaborator integration and service quality. However, in addition to the limited sample size, their research did not analyze either the degree of integration between internal integration, customer integration, and logistic collaborator integration, or the effect on SCR and company performance. Therefore, if other parts of SCR are combined and examined closely, greater insight into how integration works within SCR and its effects on sustainability.

[38] concluded that supply chain integration has a positive correlation with supply chain agility. A stable and intense supply chain integration relationship enables efficient information sharing, knowledge exchange, and production transactions between suppliers and buyers, implying a high degree of supply chain agility. With the latter, supply chain partners enjoy more extensive and transparent access to transacting information which increases the possibility of identifying supply chain risks. This facilitates their joint adaptation to and recovery from risks through enhanced collaboration. The robust supply chain system accelerates recovery from risks in comparison to the remaining three sample companies, signifying that it demonstrates the highest level of supply chain resilience. Supply chain integration provides resources such as information exchange and mutual technology-based learning to supply chain partners. Policymakers could also support the enhancement of both supply chain agility and robustness by providing other types of services [39]. Based on the literature review conducted, the following links between resource integration, collaboration, and SCR are proposed:

P1: Resource integration positively influences collaboration.

P2: Resource integration positively influences supply chain resilience.

### 2.3 Institutional arrangements

Institutions form one of the supply chain eco-systems that the government represents in regulating business competitiveness. “Institutions” are defined as the rules, norms, and beliefs created by humans which either enable or restrict action, while “institutional arrangements” refers to higher-level collections of interconnected institutions [20]. Institutional arrangements have attracted increasing attention – within SDL and service science – as a critical component of achieving resource integration, value co-creation, and service-related innovation ([40,39]). Policymakers should promote supply chain integration by eradicating factors that impede supply chain integration and by encouraging supply chain partners to strengthen their integration.

The government, as the institutional regulator with a supply chain actor role, has been primarily analyzed within the context of subsidies [41], sustainability, and reverse supply chains [42]. Companies and other competing organizations must cooperate or compete, an issue affecting fashion supply chains as demonstrated by a case study of rival companies [43]. [20] considered institutionalization as a core process of value co-creation and integral to service ecosystems. Thus, the following propositions concerning institutional arrangements and resource integration, collaboration, SCR, and sustainability are proposed:

P3: Institutional arrangements influence resource integration.

P4: Institutional arrangements influence collaboration.

P5: Institutional arrangements influence supply chain resilience.

P6: Institutional arrangements influence sustainability.

### 2.4 Collaboration

Due to globalization and amid global uncertainties, competition no longer involves individual enterprises but, rather, entire supply chains. Product manufacturers must cooperate with component suppliers throughout the supply chain in order to outperform commercial competitors. Collaboration between organizations operating within a supply chain is what connects the network as a whole and permits a holistic approach to supply chain resilience [44]. The underlying philosophy of such collaboration is that by sharing information and applying common knowledge throughout the chain, uncertainty can be reduced [45]. As a result, the recovery time, cost, disruption absorption, and ability to reduce the impact of loss can all be used to assess resilience [46]. explained how certain collaborative activities (information sharing, collaborative communication, cooperatively developed knowledge, and joint relationship initiatives) improve supply chain resilience by increasing visibility, velocity, and flexibility.

In their study [47], confirmed the value of collaboration as a response to the disruption of commercial and humanitarian supply chains and a means of re-establishing such chains. Hence, the supply chain reactive capabilities within the SCR scale are empirically validated by this research [48]. present a typology of resilience methods associated with various forms of collaboration within and between the supply networks mentioned in this article. These authors identify a third form of resilience, referred to as meso-level resilience. Therefore, the following proposition regarding collaboration, resilience and sustainability is proposed:

P7: Collaboration influences supply chain resilience.

P8: Collaboration influences sustainability.

### 2.5 Supply chain resilience (SCR) and sustainability

While the study of resilience and sustainability has distinct scope and contexts, several scholars have attempted to integrate the two in their research [49]. The nexus and links between the two themes are frequently incoherent since there is confusion over the construction of sustainable and resilient supply chains, compounded by a lack of clarity regarding which methods could benefit both sectors. A significant inherent tension exists here because sustainability is often concerned with efficiency, whereas resilience is linked with effectiveness. Research into integration, or the relationship between resilience and sustainability, remains ongoing and continues to develop [50].

In addition to these early attempts to define sustainable and resilient supply networks by combining the two notions, a lack of agreement persists among scholars as to what constitutes a sustainable and resilient supply chain. Certain researchers claim that sustainability constitutes a resilience antecedent ([51,52]) and that sustainability practices can improve resilience [53]. Approaches that improve one do not always improve the other [54]. Nevertheless, these findings are data-dependent, with the management insights being based on several simulation runs including replications. Sustainable and resilient supply networks are the most evolved forms of supply chains, as they have a wider scope and set of performance criteria. While the fundamental principles of sustainability and resilience are somewhat incompatible, there are certain overlaps in their overarching strategic aims [55]. Companies prefer to work with a supply chain partner which can manage disruptions. Customers respect a company that can rapidly overcome problems, but they also expect it to learn from its mistakes. Within this context, this research attempts to bridge this gap by combining selected parts of resilience and sustainability in terms of the supply chain. Since research into the integration of these characteristics remains in its infancy, this investigation has high novelty value and will contribute to both theory and practice. As a result, the following represents a proposal for supply chain sustainability and resilience:

P9: The influence of supply chain resilience on sustainability.

### 2.6 System theory

The tenets of SDL are viewed through an underlying theory as a means of investigating the novelty of integrating SDL premises with the theoretical foundation of SCRTS [56]. identified system theory and dynamic capability theory (DCT) as relevant in this regard. According to Ref. [20], institutional theory, systems theory, complexity theory, complexity economics, and evolutionary theory constitute the key theoretical pillars of S-D logic. Consequently, the main focus of SDL and SCRTS bridging theories was on system theory.

In the opinion of [57], a supply chain is viewed from the perspective of SD-logic as a service eco-system in which stakeholders are connected as elements of that system. Within system theory, supply chains are open and porous systems that interact with their surroundings and are, therefore, continually evolving [58]. From this perspective, it is relatively straightforward to regard supply chains as interconnected networks [59], both interacting with and relying on external inputs in addition to those from one another [60]. Another frequently applied supply chain theory that potentially constitutes a valuable lens through which to analyze data quality challenges is that of the systems theory of organizations [61]. This is because such systems are intra-organizational in nature and rely on interactions and inputs from a wide range of inter-and intra-company actors [58]. Although differences exist between physical (operand) systems and “conceptual physical” (operant) systems [62], the two groups share several characteristics. Because these discrete components are themselves sub-systems, the issue of interdependence between them also exists. The broad concept of a system border is related to this interaction between system components since in order for systems to interact with one another they require a boundary.

System theory provides a holistic and integrated perspective which aids managers and the entire organization. However, it is regarded as excessively abstract and nebulous to be applicable to real-world situations, managers are obliged to consider the organization as a whole. Consequently, rather than concentrating on individual actions, a manager might think about them collectively and develop a plan. Sub-systems are both interconnected and interdependent in character. Managers will, therefore, have a greater understanding of how disruption in one section of the company affects other parts of the business as well as the functioning of the entire organization. Unfortunately, system theory does not equip managers with actual tools and approaches, rendering its comprehensive application challenging. Nor does it say anything about how an organization interacts with and depends upon its environment.

## 3 Methodology

The involvement of the SDL and SCR within sustainability concepts represents the focus of this research. The initial step of conducting a literature review is undertaken to define the topic, followed by seeking, analyzing, and reviewing relevant data. ScienceDirect, ProQuest, and Emerald were used to search for and download papers. According to Ref. [63], systematic literature reviews involve adopting systematic, transparent and repeatable methodology to reduce bias when selecting which articles to include in or exclude from the review. Furthermore, a systematic literature review ensures a precise search approach that yields a reliable and transparent result [64]. proposed that a systematic review entails a number of steps, including establishing research questions, defining the inclusion and exclusion criteria to be adopted, explaining keyword-based search terms, compiling relevant literature, analyzing the literature selected, and defining the synthesis.

**Fig. 1:**
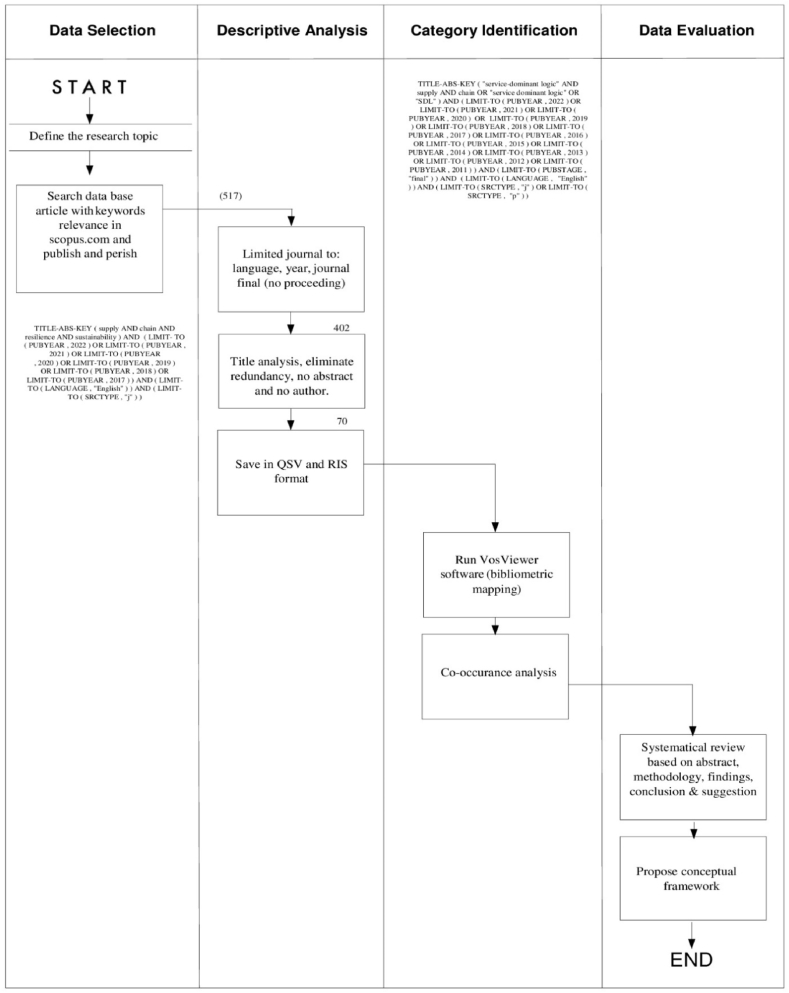
Systematical Literature Review methodology process framework [66].

[65] pointed out that because systematical literature review methodology is not standardized authors can modify it to suit their own objectives. Therefore, the literature review conducted follows the four steps as suggested by Ref. [66], namely: data selection, descriptive analysis, category identification, and data evaluation as shown in Fig. 1. However, the outcome of this literature research is a proposed conceptual framework, while the previous systematic literature review involves only a bibliometric analysis.

In the interests of reliability, only Q1, Q2, and Q3 Scopus journal indexes were selected. The topic of interest was defined on the basis of personal experience and phenomena, leading to the decision to use SD-logic and supply chain discipline. Because of the number of papers and age of the SDL concept used in the supply chain, it was deemed reasonable to search for papers dating from the previous ten years. In the scopus.com database, academic papers discussing SDL-related topics were accepted between 2004 and 2022, resulting in a corpus totaling 1953. However, only 517 papers focusing on SDL in conjunction with the supply chain were identified, a figure further reduced to 402 which met the criteria applied relating to language, year of publication, and type. Finally, an assessment of the redundancy of the contents of their abstracts eliminated all but 70 papers which were ultimately considered relevant to this study.

The search results for papers on SDL and the supply chain are saved in QSV format files for subsequent analysis using the free software tool VOSviewer for bibliometric mapping. The QSV file as the data source will be read by the same means in order to establish the occurrence of keywords in the abstract and title as well as the strength of their relatedness. The result reflected the function of VOSviewer software in displaying large bibliographic maps while providing a simple means of interpreting the results [67].

**Fig. 2:**
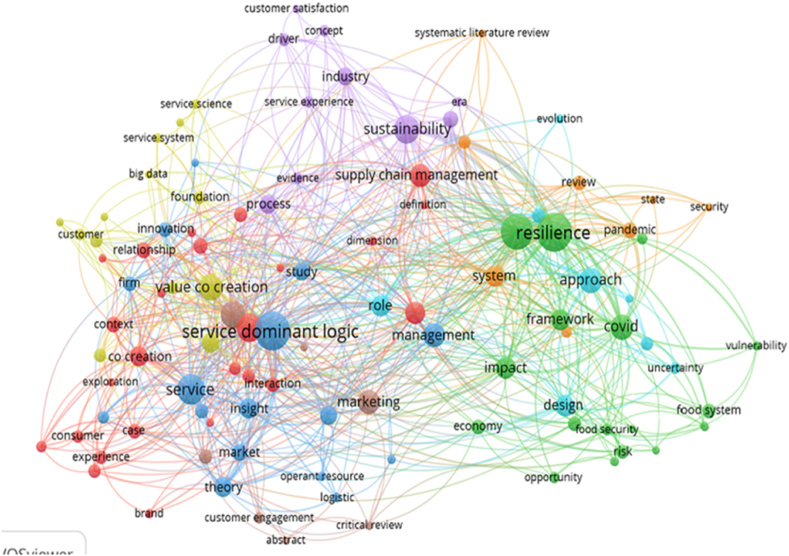
Co-occurrences analysis.

Co-occurrence analysis in VosViewer is a bibliometric analysis technique that visualizes the commonly employed and strongly correlated phrases or words discovered in publications pertaining to a certain subject of study. All keywords, including the author's name and index keywords, were subjected to a co-occurrence analysis which was used to explain the results. As illustrated in Fig. 2, a correlation between sustainability and SCR, albeit persistently tenuous in nature, indicates that this potential field of research is remains highly novel.

A review of previous academic papers identified five variables which can construct the link between SDL and SCRTS as shown in Table 2. These comprise resource integration, institutional arrangements, collaboration, supply chain resilience, and sustainability. Each variable has associated indicators, those of resources integration being internal integration, supplier development, information technology, commitment, and collaborative culture. The indicators of institutional arrangement include supply chain guidelines, centralized databases, integrity policy, tax regulation, and coordination and control, while those of collaboration comprise information sharing communication, relationships, and strategic alignment. Indicators of SCR comprise alertness, readiness, efficiency, redundancy, agility, flexibility, financial strength, responsive, recovery, and robustness. Lastly, indicators of the sustainability variable consist of environment, social, economy and reputation. This study seeks to investigate both the background and the summary, as shown in Table 2.

**Table 2:**
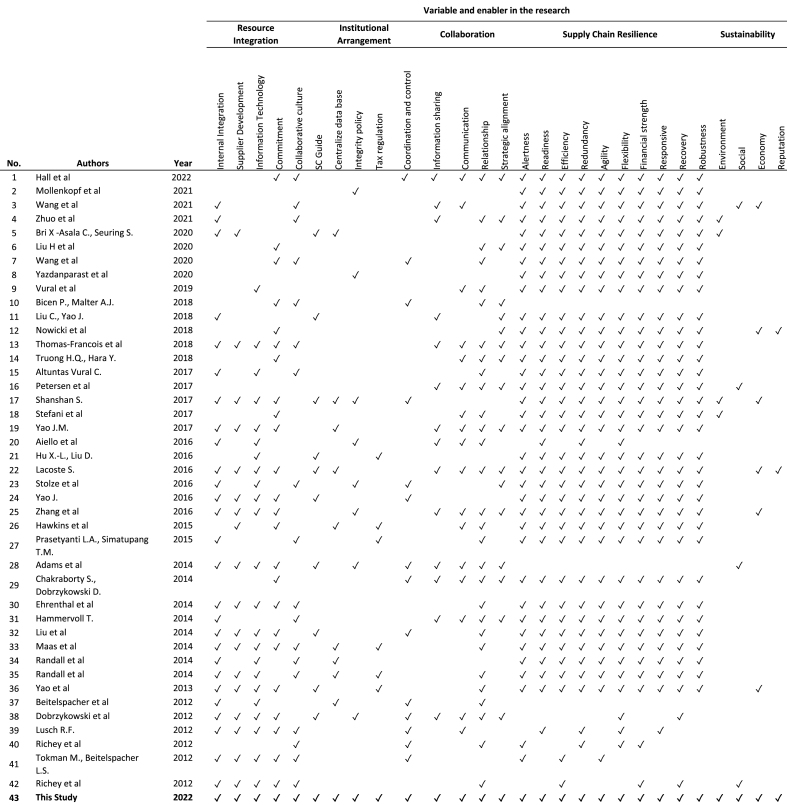
State of the art SDL and SCRTS.

Table 2 demonstrated that the study in supply chain evolved periodically and was combined with other variables that were either major or small in relation to the investigation. The papers selected from last ten years which discussed SDL and SCRTS combination. The reason to select the last ten years due to the availability of the papers regarded to this study objective.

**Fig. 3:**
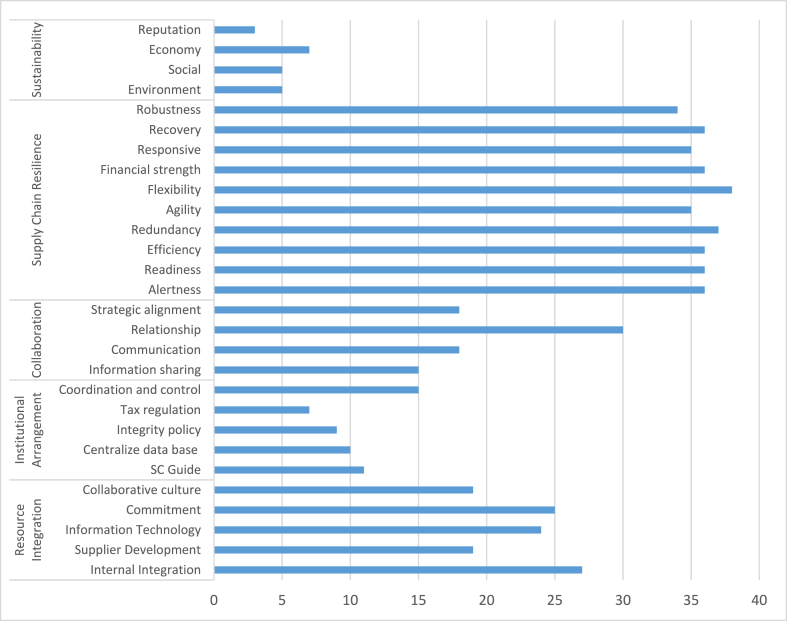
Distribution studies by enablers of SDL and SCRTS.

Additional analysis of tabel 2 as shown in Fig. 3, the majority of articles concentrate on resource integration and cooperation as enablers of SCR and SDL. While institutional arrangement and SCR are still in their infancy when paired with SCR. In light of the fact that institutional arrangement may vary by country and industry, this study identifies institutional arrangement as one of its distinctive features. Therefore, it will be interesting for future studies to empirically evaluate the role of institutional structure as a component of SDL paired with SCRTS.

In addition to the conceptual framework, there are nine hypotheses presented based on the literature review. In order to prove the hypotheses or refine the conceptual framework, empirical evidence shall be discussed further in future research. According to reference [68] there are qualitative, quantitative, and mixed methods for proving a hypothesis. In this notion, one of them or a combination of both will be suitable to the specified industrial sector. However, in this study we propose the quantitative research method, data collected thorough the questionnaires distributed to the participants and the technique of data collection in which each respondent asked to respond to the same set of questions [68]. A Likert scale is an ordered scale from which respondents choose one option that best aligns with their view [69]

The uses of a 5-point Likert scale, namely, “strongly disagree,” “disagree,” “neutral,” “agree,” and “strongly agree.” The data collected will be analyzed using SEM/SmartPLS 3.0 to test the suggested study model using partial least squares (PLS) structural equation modelling (SEM). PLS-SEM is a causal modelling technique that aims to maximize the variance explained of dependent latent components by estimating partial relationships in a series of ordinary least squares regressions [70] The decision to utilize PLS-SEM in this research was based on its capacity to manage small sample numbers.

In the conclusion section has been added about this hypothesis examination in the future research.

## 4 Results

**Fig. 4:**
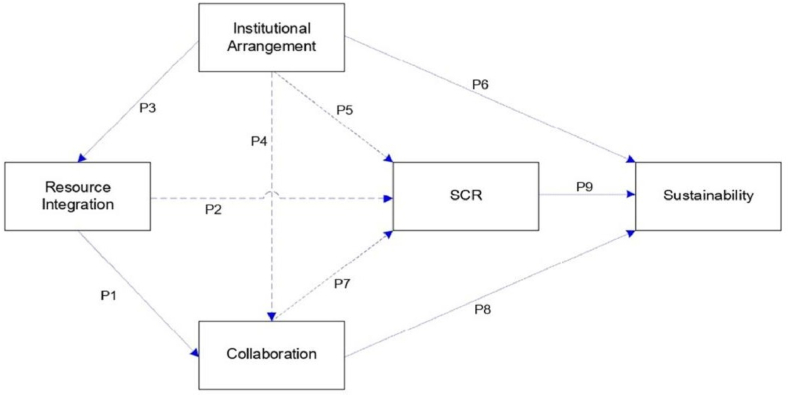
Conceptual Framework – SDL and SCRTS *(Adopted from:* [22,72,73,20,34]).

The variables and indicators of SDL and SCRTS which derived from literature review as shown in Table 2, enable the constructing of a framework necessary for an analysis of their correlation. Institutional configuration, resource integration, and collaboration serve as independent variables of SDL, while the dependent variables are SCR and sustainability. The construction of variables and indicators is based on the relevant literature and may be added or removed depending on observations made during fieldwork. However, these variables and indicators need to be supported in order to develop the framework model depicted in Fig. 4 which should be modified if any variables or indications ultimately prove to be unimportant. According to Ref. [71], there are three main approaches to performing exploratory research: completing a literature review, holding discussions with subject-matter experts, and conducting focus group interviews.

System theory, which is relevant to the concept of SDL's role in SCRTS, is the underlying theory guiding the research. However, the primary objective of this study is to propose a conceptual framework of SDL on SCRTS, taking into account particular enablement and dimensions. It is anticipated that the SCRTS drivers will be validated through this investigation, perhaps even to a larger degree than in the existing literature, by the creating of a framework based on the enabler correlation shown in Fig. 4. The framework was created by combining the earlier research conducted by Refs. [22,72,73,20,34]. Due to the numerous goals of the experiments, no single model was developed. However, depending on the objectives of the study, a specific mix of variables may be used to build the model as also depicted in Fig. 3.

Because of this, the model may be improved using real-life data to address the question of the extent to which the SDL, through its facilities, helps SCR achieve sustainability. The source and main focus of the antecedents within the context of SDL and SCRTS research is described in Table 3.

**Table 3:** Enabler of SDL and SCRTS.

No	Variable	Elements	Main Focus	Reference
1	Resource Integration	Internal integration	The degree to which a company has incorporated web-based processes for online order taking and receipt, after-sales service, and integrated demand/forecasting with customers.	Wang et al. (2021); Zhuo et al. (2021); Adams et al. (2014); Zhang et al. (2014); Tarigan et al. (2021); Chen et al. (2018); Rajesh (2018)
		Supplier development	How far a company has advanced web-based procedures with suppliers for any of the following.	Yusuf et al. (2014); Dobrzykowski et al. (2012); Adams et al. (2014); Zhang et al. (2014); Aggarwal & Srivastava (2019); Tarigan et al. (2021); Chen et al. (2018) Soni et al. (2014)
		Information Technology	To establish a competitive edge, obtain the newest technological components and offer functionalities that customers require.	Ambulkar et al. (2015); Dobrzykowski et al. (2012); Adams et al. (2014); Zhang et al. (2014); Hall et al. (2022);
		Commitment	The committed party believes the relationship lasts forever and commitment is at the heart of all relational exchanges between the company and its many patterns. Relationship s with others are so vital as to warrant making the greatest effort to sustain them.	Wu et al. (2014); Adams et al. (2014); Hawkins., (2015); Zhang et al. (2014); Chakraborty, (2018)
		Collaborative culture	A significant amount of cooperation between several parties is required. Businesses simultaneously cooperate and compete to achieve a win-win outcome.	Ambulkar et al. (2015); Dobrzykowski et al. (2012); Adams et al. (2014); Zhang et al. (2014); Aggarwal & Srivastava (2019); Kumar & Banerjee., (2012);
2	Institutional Arrangement	SC Guideline	The regulation listed all acceptable and unacceptable behaviour including, but not limited to, integrity and taxation.	Adams et al. (2014)
		Centralize data base	The quality of an organization's IT infrastructure, its IT business competence, and its level of learning determine its level of competitive advantage.	Mandal (2017)
		Integrity Policy	Highlighting the need to promote an integrity policy in supply chain operations	Beitelspacher et al. (2012)
		Tax regulation	Government financial assistance in the form of incentives, tax breaks, loans, and logistical help in the form of lenient laws and regulations is crucial, especially in unusual circumstances.	Newton et al. (2013)
		Coordination and control	Arranging and controlling systemic differences within a problem-solving procedure.	Chowdhury and Quaddus (2017); (Chounta et al., 2014; Heimeriks and Schreiner, 2002)
3	Collaboration	Information sharing	Sharing timely and accurate information reduces risk and enhances SCR capability. The necessity for a strong and specialized infrastructure supportive of effective information sharing is necessitated by the complicated nature of work. This involves a huge volume of data interchange and a highly collaborative work process between diverse parties.	Hall et al. (2022); Wu et al. (2014); Dobrzykowski et al. (2012); Adams et al. (2014); Beitelspacher et al. (2012); Aggarwal & Srivastava (2019); Soni et al. (2014); Hosseini et al. (2019); Poberschnigg et al. (2020); Hosseini, S et al. (2019); Singh et al. (2019); Rajesh (2018); Papadopoulos (2017)
		Communication	Organizations' readiness to communicate with stakeholders and discuss ideas, visions and other pertinent information in a way that satisfies their requirements.	Hall et al. (2022); Wu et al. (2014); Adams et al. (2014); Hawkins., (2015); Lacoste, (2015) (Cao and Zhang, 2011; Heimeriks and Schreiner, 2002; Meng, 2012; Palmer, 1996; Vaart et al., 2012)
		Relationship	Organizational relationships that encourage people to collaborate in achieving a similar aim.	Hall et al. (2022); Wu et al. (2014); Adams et al. (2014) (Anbanandam et al., 2011; Boeck and Wamba, 2008; Fynes et al., 2005)
		Strategic alignment	The plan is in line with the mission, objectives, goals, corporate direction, and business strategies of the company.	Hall et al. (2022); Wu et al. (2014); Adams et al. (2014), Setia and Patel (2013)
4	Supply Chain Resilience	Alertness	Focuses on the detection and monitoring of changes and measures the company's capacity to quickly identify changes in their operations.	Shin and Park (2021); Zhuo et al. (2021); Mubarik et al. (2020); Mandal (2017)
		Readiness	Supply chain disruption phases have been used to structure the adaptive resilience capability.	Grötsch et al. (2013); Mubarik et al. (2020); Sheffi, 2005
		Efficiency	The capacity to achieve results with minimal resources	Shin and Park (2021); Rajesh (2018); Chowdhury and Quaddus (2017); Pettit et al., 2010
		Redundancy	By using spare capacity in production, transportation, or inventory, redundant capacity enhances the SC's power to adjust to unanticipated disruption.	Rajesh (2018); Chowdhury and Quaddus (2017); Wieland & Wallenburg, (2012);
		Agility	More rapid responses from an agile SC enables swift adaptation to unforeseen changes in supply or demand.	Kaviani et al. (2020); Shin and Park (2021); Zanon et al. (2021); Zhuo et al. (2021); Tarigan et al. (2021); Altay., (2018); Mubarik et al. (2020); Mandal (2017)
		Flexibility	Increased adaptability promotes operating efficiency under normal circumstances and enables quick and easy modification in the event of disruption.	Kaviani et al. (2020), Shin and Park (2021); Altay., (2018); Rajesh (2018); Chowdhury and Quaddus (2017)
		Financial strength	Ability to withstand changes in financial flow	Shin and Park (2021); Wu et al. (2014); Dobrzykowski et al. (2012); Hall et al. (2022); Chowdhury and Quaddus (2017); Pettit et al., 2010
		Responsive	How quickly businesses can act on information in responding to sudden changes in supply and demand. How well-equipped are businesses to respond to customer needs?	Shin and Park (2021); Zanon et al. (2021); Zhuo et al. (2021) (Christopher and Peck 2004)
		Recovery	The ability to quickly return to a normal operational state	Shin and Park (2021); Zhuo et al. (2021); Aggarwal & Srivastava (2019); Chowdhury and Quaddus (2017); Pettit et al., 2010
		Robustness	Ability to perform its role and dampen unexpected disturbance. If any changes take place, a sturdy SC maintains its stability and withstands shock.	Zhuo et al. (2021); Rajesh (2018); Wieland & Wallenburg, (2012);
5	Sustainability	Environment	Stakeholders should take the necessary steps to reduce the environmental impact of their deliverables. Stakeholders should take pro-active measures to protect the environment as a whole.	Wang et al. (2021); He et al. (2020); Tarigan et al. (2021); Gouda & Saranga, (2018);
		Social	To prevent harm from being done to society	Yusuf et al. (2014); He et al. (2020); Tarigan et al. (2021); Rajesh (2018); Gouda & Saranga, (2018);
		Economy	To avoid penalties or other losses caused by unreliable products, it is important to verify that products comply with environmental laws.	Newton et al. (2013); Adams et al. (2014); Hall et al. (2022); Tarigan et al. (2021); Gouda & Saranga, (2018);
		Reputation	The degree to which customers consider a business trustworthy and concerned about them. Value is created through interactions both within and between relationships.	Tieman (2017); Liu et al. (2021); Quintana- García et al. (2021) (Doney and Cannon, 1997). Vargo and Lusch (2008)

## 5 Conclusion

In an era of disruption, supply chain sustainability is essential to maintaining competitive advantage. In order to be competitive and build resilience, the SDL perspective recommends value co-creation between actors. A number of previous studies have attempted to interconnect the SDL concept with research into the supply chain. However, this study is the first conceptual framework constructed by means of a literature review that combines SDL and SCRTS. This study identified 42 relevant papers and examined which of them explicitly discussed the correlation between SDL and supply chain management research. The literature review highlighted five variables and 28 indicators applicable to an exploration of the relationship between SDL and SCRTS. However, despite the continuing fact that few academic papers have looked at SDL and SCRTS, research focusing on SDL in supply chains has progressed. The difficulty in applying supply chain research in isolation without combining it with SDL represents one reason why there has been no rapid proliferation in the number of articles. “Are we there yet?” asked [27]. These academics’ conclusions indicate that SDL is reliable as a performance-based logistic.

The framework has been carefully analyzed and selected in accordance with the concepts of SDL and SCRTS. In order to develop an understanding of how SDL affects SCRTS over time, future research to proove the hyprotheses could use this study as the starting point for an analysis of the literature on SDL and SCRTS and for putting these concepts to the test within any industry. Moreover, future research into how the SDL concept can be applied to particular premises and practically tested using sufficient data. The methodology may be qualitative, quantitative, or a combination of the two to prove the hypotheses in the industry of interest. While the proposed conceptual framework is general and applicable to various sectors, it may be modified on the basis of actual SDL and SCRTS research which remains in its early stages.

This study has broadened the time frame of the academic papers used as reference sources to the last ten years. However, one limitation has proved to the limited number of such sources which specifically discuss SDL and supply chain management. Moreover, the review consisted of a keyword search which restricted the results to phrase combinations and it is possible that the keywords used in the conduct of this study are far from exhaustive.

## Author contribution statement

All authors listed have significantly contributed to the development and the writing of this article.

## Funding statement

This research did not receive any specific grant from funding agencies in the public, commercial, or not-for-profit sectors.

## Data availability statement

No data was used for the research described in the article.

## Declaration of interest’s statement

The authors declare no conflict of interest.
